# Multidimensional MRI-CT atlas of the naked mole-rat brain (*Heterocephalus glaber*)

**DOI:** 10.3389/fnana.2013.00045

**Published:** 2013-12-20

**Authors:** Fumiko Seki, Keigo Hikishima, Sanae Nambu, Kazuo Okanoya, Hirotaka J. Okano, Erika Sasaki, Kyoko Miura, Hideyuki Okano

**Affiliations:** ^1^Department of Physiology, Keio University School of MedicineTokyo, Japan; ^2^Central Institute for Experimental AnimalsKanagawa, Japan; ^3^Laboratory for Symbolic Cognitive Development, RIKEN Brain Science InstituteSaitama, Japan; ^4^Japan Science and Technology Exploratory Research for Advanced Technology Okanoya Emotional Information ProjectSaitama, Japan; ^5^Department of Life Sciences, Graduate School of Arts and Sciences, The University of TokyoTokyo, Japan; ^6^Division of Regenerative Medicine, Jikei University School of Medicine, Tokyo, Japan; ^7^Precursory Research for Embryonic Science and Technology, Japan Science and TechnologySaitama, Japan; ^8^Riken Keio University Joint Research Laboratory, RIKEN Brain Science InstituteSaitama, Japan

**Keywords:** cortical thickness, diffusion tensor imaging, eusociety, MR histology, postnatal development, rodents, sensory system, social behavior

## Abstract

Naked mole-rats have a variety of distinctive features such as the organization of a hierarchical society (known as eusociality), extraordinary longevity, and cancer resistance; thus, it would be worthwhile investigating these animals in detail. One important task is the preparation of a brain atlas database that provide comprehensive information containing multidimensional data with various image contrasts, which can be achievable using a magnetic resonance imaging (MRI). Advanced MRI techniques such as diffusion tensor imaging (DTI), which generates high contrast images of fiber structures, can characterize unique morphological properties in addition to conventional MRI. To obtain high spatial resolution images, MR histology, DTI, and X-ray computed tomography were performed on the fixed adult brain. Skull and brain structures were segmented as well as reconstructed in stereotaxic coordinates. Data were also acquired for the neonatal brain to allow developmental changes to be observed. Moreover, *in vivo* imaging of naked mole-rats was established as an evaluation tool of live animals. The data obtained comprised three-dimensional (3D) images with high tissue contrast as well as stereotaxic coordinates. Developmental differences in the visual system were highlighted in particular by DTI. Although it was difficult to delineate optic nerves in the mature adult brain, parts of them could be distinguished in the immature neonatal brain. From observation of cortical thickness, possibility of high somatosensory system development replaced to the visual system was indicated. 3D visualization of brain structures in the atlas as well as the establishment of *in vivo* imaging would promote neuroimaging researches towards detection of novel characteristics of eusocial naked mole-rats.

## INTRODUCTION

Naked mole-rats (*Heterocephalus glaber*), small fossorial rodents that live underground in the Horn of Africa (Somalia, Ethiopia, and Kenya), have been studied by researchers because they possess several unique biological characteristics. They are famous for their hyper-longevity: although the average body length of a naked mole-rat is only 8 cm, their average lifespan is approximately 28 years (Buffenstein and Jarvis, 2002; Buffenstein, 2005, 2008; Edrey et al., 2011). They show considerable resistance to cancer (Buffenstein, 2005), are insensitive to pain caused by certain chemicals (capsaicin and acid; Park et al., 2008), and are tolerant to hypoxia (Larson and Park, 2009). Also, they are almost blind and are one of only two known eusocial mammals. They form underground colonies averaging 60–80 individuals. Their society consists of a single breeding female (queen), one to three breeding males (king(s)), and numerous non-reproductive subordinates (Jarvis, 1981). Subordinates do not show mating behaviors and act as “workers” or “soldiers” even when they have reached adulthood. The reproduction of subordinates is suppressed and they are instead engaged in foraging, maintaining the nest, caring for the young, and defending the colony from invasion by foreign enemies (Sherman et al., 1991).

To examine such unique and interesting features, detailed investigation of brain structures is of importance. The primary step is to establish the databases for anatomical characterization. The first atlas of naked mole-rats (Xiao et al., 2006) presents cytoarchitectural information with anatomical labeling, which have been widely used as the gold standard. However, the spatial relationships also need to be documented considering that brain structures are composed of three-dimensional (3D) organization. Magnetic resonance imaging (MRI) can resolve this issue because 3D images with high spatial resolution can be generated in a non-invasive fashion. MR histology techniques enable to generate 3D acquisition with isotropic voxels of several tens of micrometers (Petiet et al., 2008). The data are digitized, which allows them to be manipulated with ease, making it possible to perform the variety of image analyses unique to MRI atlases. Examples include an MRI mouse brain atlas combined with computed tomography (CT; Chan et al., 2007) and an MRI stereotaxic developmental brain atlas combined with diffusion tensor imaging (DTI) and CT (Aggarwal et al., 2009). An advantage of using non-invasive MRI and CT is to easily construct stereotaxic coordinates by combination of modalities without shrinkage of a specimen, which is difficult with histological methods. DTI, an advanced MRI technique, is capable of visualizing white matter fiber structures through analysis of the directional diffusion of water molecules within nerve tissue (Mori et al., 2001; Zhang et al., 2003). It enables researchers to evaluate neural fiber connectivity, which is a labor intensive exercise when performed using other techniques such as neuronal fiber tracers or tract-selective immunohistochemical staining (Fujiyoshi et al., 2007). Hence, MRI can provide a wealth of anatomical information through its capacity to depict the brain from multiple perspectives, such as in terms of neural morphometry and connectivity.

The aim of this study was to use these advanced MRI techniques to reveal the neuroanatomical structure of the naked mole-rat brain. Visualizing the shapes of brain structures and white matter bundles in 3D for the first time would allow us to highlight the diverse characteristics of this species, and might provide information about their unique sensory system and social system. The atlas would also contain the same kind of anatomical information from the neonatal brain MRI, making it possible to observe developmental changes. Furthermore, *in vivo* imaging was established, which was the critical step to elucidate a mechanism of brain development and a process of sexual maturation in future.

## MATERIALS AND METHODS

### DATA ACQUISITION OF THE ATLAS

All procedures were performed in accordance with the Laboratory Animal Welfare Act and the Guide for the Care and Use of Laboratory Animals (National Institute of Health, Bethesda, MD, USA). All experiments were approved by the Animal Study Committee of Keio University in Japan (approval number; 09212-(1)). A male adult breeder (6 years old), a neonatal naked mole-rat (postnatal day 8), and a mouse (1 year old) for each were euthanized under deep anesthesia with isoflurane (Abbott Laboratories, Abbott Park, IL, USA) and chemically fixed with phosphate-buffered saline containing 4% paraformaldehyde for 2 weeks. The skull was first imaged using a CT scan (R_mCT2, Rigaku Corp., Tokyo, Japan). With peak voltages of 90 kV, whole skull data were obtained within 2 min with isotropic resolution of 100 μm^3^. This procedure was performed on the skull of the adult naked mole-rat. The specimens were then soaked in phosphate-buffered saline containing 0.5% sodium azide and the contrast agent gadopentetate dimeglumine (1 mM Magnevist; Schering, Berlin, Germany) for another 2 weeks to increase MRI tissue contrast. They were positioned in an acrylic tube filled with fluorinert (Sumitomo 3M Limited, Tokyo, Japan) to diminish the signal intensity due to the medium around the specimens. Brain MRI data were acquired using a 7T Biospec 70/16 scanner with CryoProbe (Bruker biospin MRI GmbH; Ettlingen, Germany). 3D ultra-high resolution T_2_-weighted images (T_2_WI) with isotropic resolution of 35 μm^3^ were obtained by rapid acquisition with relaxation enhancement (RARE) with the following parameters: repetition time, 600 ms; echo time, 16 ms; number of averages, 1; and RARE factor, 2.

The next step was to acquire 3D ultra-high resolution DTI data with isotropic resolution of 67 μm^3^ using a Stejskal–Tanner diffusion spin-echo sequence with the following parameters: repetition time, 600 ms; echo time, 28 ms; number of averages, 1; *b*-value, 2000 s/mm^2^; number of motion probing gradient orientations, 12 axes. **Table 1** shows our normalized diffusion gradient orientations. The total time taken to acquire data in each scan was approximately 20 h.

**Table 1:** Normalized diffusion gradient orientations.

Image volume	Gradients	
	*x*	*y*	*z*
1	0.0000	0.0000	0.0000
2	0.8944	0.0000	0.8944
3	0.0000	0.4472	0.8944
4	0.4472	0.8944	0.0000
5	0.8944	0.4472	0.0000
6	0.0000	0.8944	0.4472
7	0.4472	0.0000	0.8944
8	0.8944	0.0000	-0.4472
9	0.0000	-0.4472	0.8944
10	-0.4472	-0.8944	0.0000
11	0.8944	-0.4472	0.0000
12	0.0000	0.8944	-0.4472
13	-0.4472	0.0000	0.8944

### DATA ANALYSIS OF THE ATLAS

Data on the skull and brain regions were semi-automatically extracted from the MRI and CT data using the “Segmentation Editor” in Amira software version 5.4 (Visage Imaging, Inc., San Diego, CA, USA). The skull was manually adjusted with affine transformation including translation and rotation by referring to stereotaxic coordinates as described in the stereotaxic mouse atlas (Franklin and Paxinos, 2007). Then, T_2_WI and DTI were fused to the skull CT with stereotaxic coordinates by rigid body registration using “Affine registration” in Amira.

A DTI direction-encoded color (DEC) map was computed and fiber structures were tracked based on the FACT-like stream line algorithm (Mori et al., 1999) by the Diffusion Toolkit and TrackVis software (Martinos Center for Biomedical Imaging, Massachusetts General Hospital)^1^ with the following parameters: fiber turning angle for ending tracking, 35°; rendering with line mode. The diffusion tensor can be represented as an ellipsoid, where a proton at the center of the voxel has an equal probability of diffusing to any point in that ellipsoid. For delineation of specific bundles by tractography, selective bundles were segmented by manually placing regions of interest with reference to the newly generated DEC map and the mouse brain atlas (Franklin and Paxinos, 2007) with multi-ROI approach (Conturo et al., 1999; Huang et al., 2004). Major brain regions (caudate putamen, hippocampus, and cerebellum) were semi-automatically segmented using the “Segmentation Editor” and reconstructed using the 3D rendering module based on polygon-based rendering in Amira. For reference, volumes of all segmented regions in both the adult and neonatal brain, as well as the whole brain volumes of brain *in vivo* were calculated the sum of tetrahedral joining surface triangles to the origin using “surface area” measurements in Amira (Brandt et al., 2005; Ullmann et al., 2010; Hikishima et al., 2011).

Three-dimensional visualization made it possible to conduct image analysis focusing on cortical distribution. To identify any features specific to naked mole-rats, the MRI of the adult mouse brain was used for comparison. The cerebral cortex of naked mole-rats (adult and neonatal) as well as those of the mouse were semi-automatically segmented with reference to atlases of naked mole-rats (Xiao et al., 2006) and mice (Franklin and Paxinos, 2007), respectively. Cortical thickness was computed from the 3D cortical area. Mapping of cortical thickness was performed by computing the distance from the vertex to the point where the normal intersected the closest triangle in each vertex on the segmented cortical surface (Zhang et al., 2003; Huang et al., 2009). Knowledge of localized cortical areas was available for the mouse brain; information about the cortical map specific to the sensory system was thus added to the resulting 3D image by manual drawing, with references to the 3D atlases (Hjornevik et al., 2007; Lein et al., 2007; Hawrylycz et al., 2011), and the mouse brain atlas (Franklin and Paxinos, 2007).

### DATA ACQUISITION OF *IN VIVO* IMAGING

*In vivo* imaging was performed on 4 naked mole-rats in a colony; 5-years-old female breeder; 41 g, 5-years-old male breeder; 40 g, 1-year old two male non-breeders 28, 38 g, respectively. Naked mole-rats were anesthetized with a mixture of 2.0% of isoflurane and oxygen at a constant rate (Herold et al., 1998) using a ventilator, SN-480-7 (Shinano, Tokyo, Japan). Both rectal and skin temperatures were monitored considering that naked mole-rats were heterothermic animal. Their temperatures were maintained around 29–32 centigrade, which was the appropriate condition for resting states of naked mole-rats (Jarvis, 1978). When their temperatures were declined, a hot-air type heater was put on for several minutes. Moisturizing gel was covered on the dorsum to prevent from drying.

An imaging coil with inner diameter of 38 mm was fitted to their head size was thus selected for *in vivo* MRI. The same sorts of image contrast with the atlas were acquired: T_2_WI with coronal section by RARE with the following parameters: repetition time, 3000 ms; echo time, 34 ms; number of averages, 25; and RARE factor, 8 and respiratory gating, and DTI with coronal section by eight-shots echo planner imaging sequence based on the Stejskal–Tanner diffusion preparation with following parameters: repetition time, 3000 ms; echo time, 41 ms; number of averages, 4; *b*-value, 1000 s/mm^2^; number of motion probing gradient orientations, 12 axes, and respiratory gating. The resolution of acquired images was as follows: T_2_WI: in plane resolution, 125 μm^2^; slice thickness, 500 μm; DTI: in plane resolution, 125 μm^2^; slice thickness, 750 μm. The total time required for an experiment was 1.5 h. Acquired data were computed in the same fashion as the atlas.

## RESULTS

**FIGURE 1:**
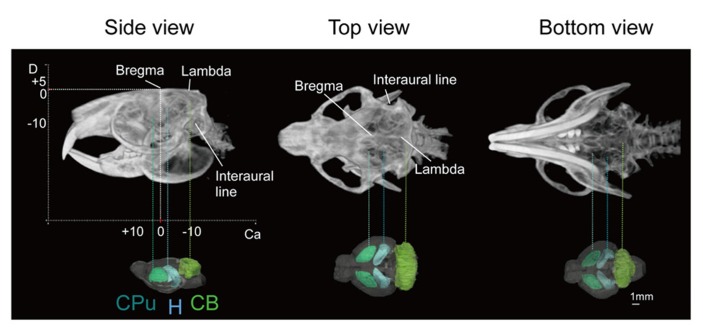
**Volume-rendered skull images with stereotaxic coordinates (left, side view; center, top view; right, bottom view) together with whole brain images displaying the approximate locations of major regions caudate putamen (CPu, light green), cerebellum (CB, green), and hippocampus (H, light blue)**. Scale bar = 1 mm.

Images of the skull of the adult naked mole-rat were first acquired with a CT scan. **Figure 1** shows a volume-rendered skull image with stereotaxic coordinates for the landmarks Bregma and lambda, and the interaural line, along with 3D brain images to reveal the approximate locations of major structures. It demonstrates that the skull was adjusted according to stereotaxic coordinates; the side view confirmed that the Bregma was parallel to the lambda.

**FIGURE 2:**
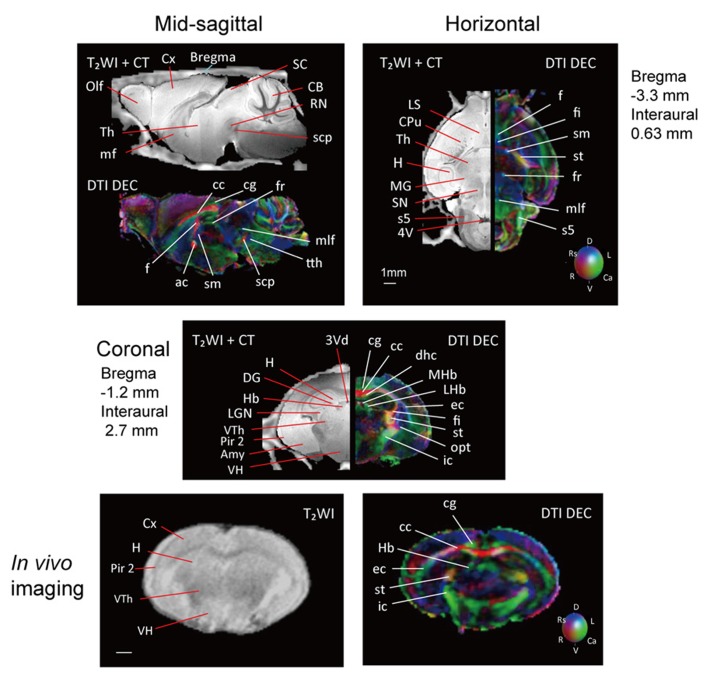
**T_**2**_WI and DTI.** One slice from the atlas: the sagittal (upper left), horizontal (upper right), and coronal planes (center). One coronal slice from *in vivo* T_2_WI (lower left) and DTI (lower right). Orientation is coded by color as follows: red is medial–lateral, green is rostral–caudal, and blue is dorsal–ventral. Scale bar = 1 mm.

**Figure 2** shows slices in sagittal, horizontal, and coronal planes from high resolution T_2_WIs (i.e., MR histology) and the DTI DEC maps with labeling of major structures. High soft-tissue contrast made various anatomical structures visible in the images. Both sets of data were combined with the skull CT data, so that the brain data included stereotaxic coordinates, as demonstrated by the MR histology. In the DTI data, visualization with different colors made it possible to localize white matter bundles, such as the medial and lateral parts of the habenular commissure, which could barely be discriminated in the MR histology coronal slices. The alphabetical listing of all abbreviations with corresponding structures is available in **Table 2**. The study focused on delineating the visual system by DTI, taking into consideration the fact that naked mole-rats are almost blind. In the brain of the adult naked mole-rat, the lateral geniculate nucleus (LGNs) and the optic tract could be identified from the coronal plane of the 2D DTI DEC map. The data presented are stored in Nifti compressed format^2^ and can be downloaded from the CT-MRI naked mole-rat brain database^3^.

**Table 2:** Alphabetical lists of all abbreviations with corresponding structures; left, T_**2**_WI; right; DTI.

Abbreviation	T_**2**_WI Full name of structures		DTI
3Vd	Dorsal third ventricle	ac	Anterior commissure
4V	Fourth ventricle	cc	Corpus callosum
Amy	Amygdala	cg	Cingulum
CB	Cerebellum	dhc	Dorsal hippocampal commissure
CPu	Caudate putamen	ec	External capsule
Cx	Cerebrum	f	Fornix
DG	Dentate gyrus	fi	Fimbra of the hippocampus
H	Hippocampus	fr	Fasciculus retroflexus
Hb	Habenular nucleus	Hb	Habenular nucleus
LGN	Lateral geniculate nucleus	ic	Internal capsule
LS	Lateral septal nucleus	LHb	Lateral habenular nucleus
LV	Lateral ventricle	MHb	Medial habenular nucleus
mf	Medial forebrain bundle	mlf	Medial longitudinal fasciculus
MG	Medial geniculate nucleus	opt	Optic tract
Pir 2	Piriform cortex layer 2	pc	Posterior commissure
Olf	Olfactory bulbs	s5	Sensory root of the trigeminal nerve
RN	Red nucleus	scp	Superior cerebellar peduncle
s5	Sensory root of the trigeminal nerve	sm	Stria medularis
SC	Superior colliculus	st	Stria terminalis
scp	Superior cerebellar peduncle	tth	Trigeminothalamic tract
SN	Septal nucleus		
Th	Thalamus		
VH	Hypothalamus, ventral parts		
VTh	Thalamus, ventral parts		

Also, **Figure 2** shows slices in coronal plane from *in vivo* T_2_WIs and the DTI DEC maps of the male breeder with labeling of major structures. Images demonstrated that the major regions could be sufficiently identified from *in vivo* T_2_WIs and DTI DEC maps. Piriform cortex could be particularly visible in the coronal plane of the T_2_WIs. In the DTI DEC maps, cingulum and external capsule could be identified. The volumes of whole brain from four individual images are shown in **Table 3**.

**Table 3:** Volumes of the whole brain in male adult and neonatal postmortem specimens, and four subjects; a female adult breeder (queen), a male adult breeder (king), two male non-breeders (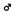
worker).

	Adult	Neonate	Queen	King	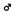 Worker	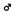 Worker
Volume (mm^3^)	484.9	249.0	484.1	493.1	489.6	484.1

**FIGURE 3:**
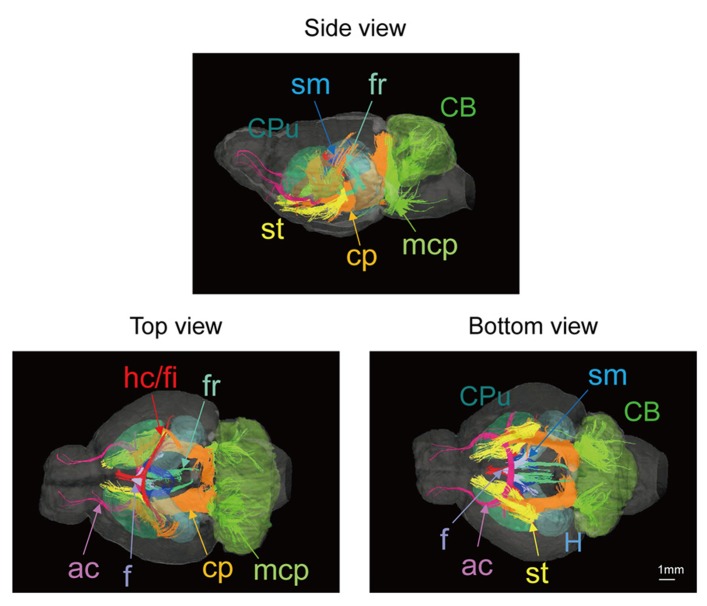
**Tractography showing the white matter tracts in 3D (upper, side view; lower left, top view; lower right, bottom view).** ac, anterior commissure (pink); cp, cerebral peduncle (orange); f, fornix (light purple); fr, fasciculus retroflexus (light green); hc/fi, hippocampal commissure/fimbria (red); mcp, middle cerebellar peduncle (green); sm, stria medularis (blue); st, stria terminalis (yellow) together with the caudate putamen (CPu; light green) cerebellum (CB; green), and hippocampus (H; light blue). Scale bar = 1 mm.

**Figure 3** shows 3D images of major white matter tracts obtained with DTI tractography (side, top, and bottom views); the structures have been colored to make them easy to identify. This approach made it possible to capture the spatial relationships between white matter tracts to detect possible neural connections between regions. 3D delineation of optic nerve was difficult while the optic tracts could be drawn with DTI tractography.

**FIGURE 4:**
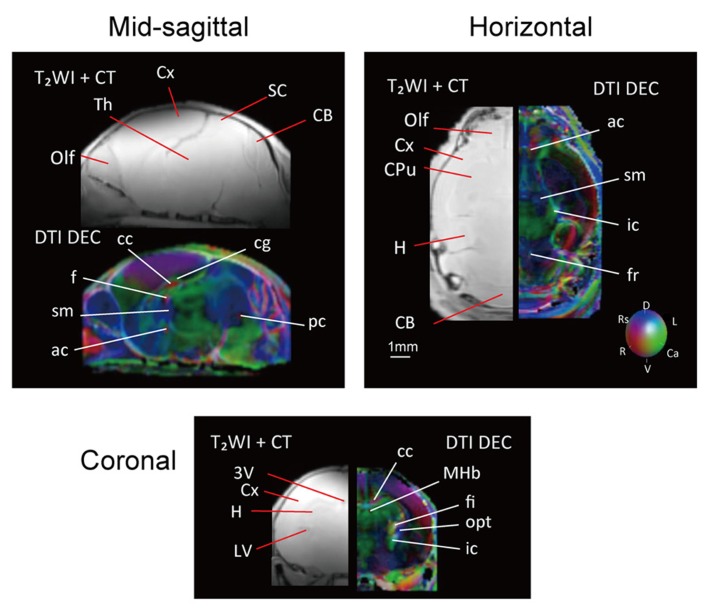
**T_**2**_WI and DTI of the neonatal brain in three different planes; the sagittal (upper left), horizontal (upper right), and coronal planes (center).** Orientation is coded using the same color scheme as in the adult brain: red is medial–lateral, green is rostral–caudal, and blue is dorsal–ventral. Scale bar = 1 mm.

The series of data obtained with the adult brain were also acquired for the neonatal brain, as shown in **Figure 4**. Neonatal brain images in the form of both MR histology and a DTI DEC map were shown in the same layout as for the adult; representative one slice each from sagittal, horizontal, and coronal planes with labeling for comparison. The contrast of MR histology was poor due to the lack of myelination compared with the adult brain. Although there were far fewer identifiable structures in the neonatal brain than the adult brain, the commissural tracts could be seen in the sagittal plane of the DTI DEC map.

**FIGURE 5:**
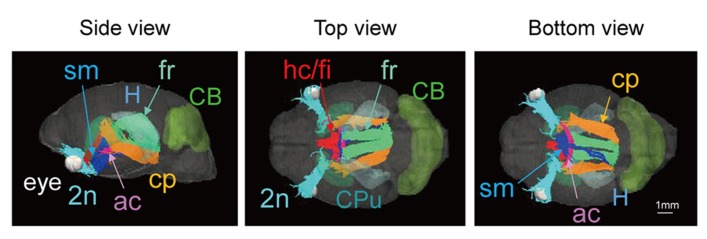
**Tractography of the neonatal brain (left, side view; center, top view; right, bottom view).** 2*n*, optic nerve (light blue); ac, anterior commissure (pink); cp, cerebral peduncle (orange); fr, fasciculus retroflexus (light green); hc/fi, hippocamal commissure/fimbria (red); sm, stria medularis (blue), together with the caudate putamen (CPu, light green), the cerebellum (CB, green), the hippocampus (H, light blue), and the eye (white). Scale bar = 1 mm.

Images from DTI tractography of the neonatal brain are shown in **Figure 5** as side, top, and bottom views. The same color scheme was applied to the neonatal brain as was used for the adult brain. While the shape of the neonatal brain as a whole shows that it is in the process of growing, the shapes of the hippocampus and the caudate putamen seemed similar to those of the adult, respectively. The volumes of segmented regions were easily measurable due to the acquisition of 3D digital data. **Table 4** shows the volumes of five major regions of both adult and neonatal brains, and suggests that the degree of development might differ depending on the region. This could also apply to delineated white matter structures: white matter tracts of the cerebral peduncle were delineated but those of the middle cerebellar peduncle were not. The development of the visual system can also be compared on the basis of its degree of delineation. Interestingly, as in the adult brain, the optic tract and LGN could be identified in the DTI DEC map of the neonate; however, it was only in the neonatal brain that the optic nerve (2*n*), which was colored light blue, could be delineated before crossing at the chiasm.

**Table 4:** Volumes of the whole brain, cerebral cortex, cerebellum, caudate putamen, and hippocampus in the adult and neonatal naked mole-rat.

Structure	Adult (mm^3^)	Neonate (mm^3^)
Whole brain	484.9	249.0
Cerebral cortex	110.4	74.1
Cerebellum	57.9	16.6
Caudate putamen	16.9	7.3
Hippocampus	13.1	3.9

**FIGURE 6:**
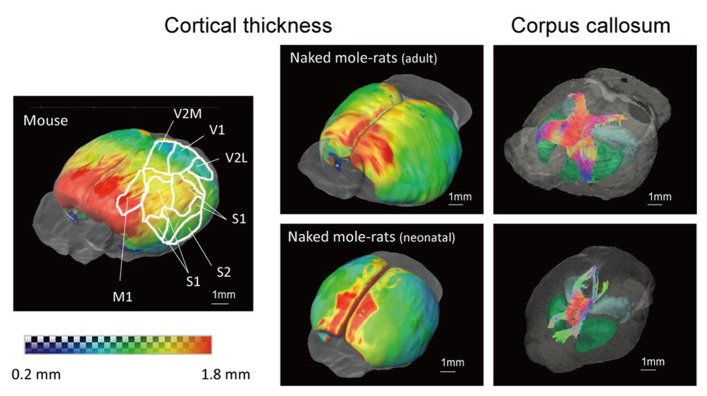
**3D surface maps showing cortical thickness and images of corpus callosum of the adult (above right) and neonatal (below right) naked mole-rats brain, and the mouse brain that maps major sensory systems (left).** Thickness is indicated by color as follows: (dark blue, 0.2 mm to red, 1.8 mm). Whole brain images with the corpus callosum are shown to the right. Colors reveal the directions of the tracts (red, right–left; green, cranial–caudal; blue, ventral–dorsal). M1, primary motor cortex; S1, primary somatosensory cortex; S2, secondary somatosensory cortex; V1, primary visual cortex; V2M, secondary visual cortex medial area; V2L, secondary visual cortex lateral area. Scale bar = 1 mm.

**Figure 6** shows 3D images of cerebral cortical thickness in the mouse brain overlaid with a cursory drawing that maps the major sensory systems, which allows comparison with images of the adult and neonatal naked mole-rat brain that were analyzed in the same fashion. The degree of cortical thickness is represented by color. Dark blue corresponds to 0.2 mm (thinnest), while red corresponds to 1.8 mm (thickest). The distribution of cortex was similar in the adult naked mole-rat and the mouse in that the rostral areas were thick; however, the caudal areas, which the visual cortex occupies, were thicker in the naked mole-rat than in the mouse. In addition, images of the corpus callosum (cc) were added to the images of the adult and neonatal naked mole-rat brain to determine whether cortical development corresponds to the development of the cc. In the adult brain, the fiber structures of the cc innervated both sides of the cortex. By contrast, in the neonatal brain, the connection was immature and projections to rostral as well as caudal areas were observed.******

## DISCUSSION

We constructed a 3D MRI-CT brain atlas and *in vivo* imaging for neuroimaging research of the naked mole-rat from multiple perspectives. Three-dimensional MR histology were used to visualize anatomical features by means of high soft-tissue contrast, representing an addition to the histological atlas (Xiao et al., 2006). The DTI DEC maps emphasized fiber bundles, and the spatial relationship between the white matter tracts and major brain regions was demonstrated by DTI tractography. Also, our datasets were easily incorporated into 3D reconstructions, which meant that we could observe differences in the distribution of cortical thickness between the naked mole rat and the mouse. Combining skull CT images with brain images means that our atlas contains stereotaxic coordinates with contrast between bone and brain tissues. This visualization will be valuable when precise targeting procedures of the brain (e.g., electrode implantation and injection of chemical substances) are required (Aggarwal et al., 2009). The anatomical information acquired will also provide information about the morphology of the sensory and social system, which could possibly be developed further.

### THE DEVELOPMENT OF THE MAJOR WHITE MATTER TRACTS AND THE VISUAL SYSTEM

It is possible to compare the development of segmented white matter tracts by referring to the mouse MRI atlas (Chuang et al., 2011). By 8 days after birth, the volume of the major white matter tracts had reached 50% of their estimated volume in the adult. The tracts include the hippocampal commissure, anterior commissure, cc, fornix, stria medularis, and fasciculus retroflexus, all of which could be delineated by tractography (Chuang et al., 2011). Given that the neonatal brain of the naked mole-rat was examined 8 days after birth, at a similar time point to that of the mouse, there may not be any obvious differences in segmented fiber structures; all white matter tracts except the fornix could also be delineated in the naked mole-rat.

On the other hand, the optic nerve in the mouse could be clearly visualized from embryonic day 12 (Chuang et al., 2011), whereas it was unclear either in neonatal or adult stages of naked mole-rats. A previous study has confirmed that the visual system of the naked mole rat has regressed due to their underground life (Hetling et al., 2005), yet it is uncertain from previous findings to what degree the naked mole-rat may retain a visual system. Despite lacking the ability to recognize shapes, which requires higher-order function, naked mole-rats have retained the muscular function of the iris compared with other genera of mole rats, suggesting that they have some ability to detect light (Nikitina et al., 2004). The presence of the olivary pretectal nucleus (Crish et al., 2006a) as well as differences in locomotor activity (due to circadian rhythms) during exposure to periodic light/dark changes (Riccio and Goldman, 2000a,b) has also been confirmed in naked mole-rats.

The optic tracts could be delineated; however, the optic nerve could not be delineated by DTI, indicating that the visual system of the adult naked mole-rat has undergone regression. On the other hand, parts of the optic nerve and the optic tract could be visualized in the neonatal brain. This implies that the development of the optic nerve may have some unique features; indeed, postnatal development of the eye is considered to be irregular, especially in the case of the lens given loss of crystallins in lens of adults which was present in neonatal were suggested (Nikitina et al., 2004).

The possibility that naked mole rats retain a visual system to some degree is also of interest, in particular whether the development of the visual system may show plasticity in response to exposure to different amounts of light after birth (Daw, 2006). Naked mole-rats in our laboratory were raised in an environment in which a certain amount of light is always present. Modifications such as an increase in brightness or limiting light exposure to 12 h per day may affect the development of the visual system. If parts of the visual system function, light stimuli may affect the development of not only the visual system but also brain structures associated with visual processing such as the visual cortex, LGN, and SC.

Magnetic resonance imaging would make it possible to follow the development of the visual system because it would permit continuous observation of the same subject. In fact, atlases of the developing brain in both rodents (Petiet et al., 2008; Aggarwal et al., 2009; Chuang et al., 2011) and non-human primates (Hikishima et al., 2013) have been available. Evaluation of developmental process in naked mole-rats may be important for understanding brain development and the visual system, and for investigating how the optic nerve develops.

In connection with this, the visual system can also be examined with respect to cortical distribution. The primary motor cortex (M1) is the thickest, while the primary visual cortex (V1) is the thinnest in humans, non-human primates, and rodents (Brodmann, 2006). M1 in both mouse and the naked mole-rat are the same distribution, but the cortex in the caudal area occupied by visual cortex seemed to be thicker in the naked mole-rat than in the mouse, according to a number of mouse anatomic atlases (Franklin and Paxinos, 2007; Hjornevik et al., 2007; Lein et al., 2007; Hawrylycz et al., 2011). This contradiction may be linked with data indicating that the somatosensory cortex has replaced significant parts of the visual cortex (Catania and Remple, 2002). Precise localization of cortical thickness would enable researchers to clarify the extent to which cortical distribution is unique such as in visual regions. It may also be possible to correlate the development of the cerebral cortex and the fiber bundles extending to the cortex, given that the cc and visual cortex reportedly show plasticity in response to visual stimuli (Pietrasanta et al., 2012).

For further investigation towards revealing morphological properties discussed in this study, it is essential to obtain more precise anatomical information of the naked mole-rat brain, considering that the data of the present atlas were limited to a single specimen. One reason for this, which we are currently trying to address, is the difficulty of securing greater numbers of naked mole-rats due to their slow reproduction. This atlas does not reflect individual differences such as sex differences or indeed the presence/absence of breeding ability, although there is reportedly no significant variation in brain weight (Xiao, 2007) or body weight (Pinto et al., 2010) between male breeders and non-breeders. The development of a population-averaged atlas using *in vivo* imaging, as has been done for other model animals (Black et al., 2001; Watanabe et al., 2001; Hikishima et al., 2011) is the crucial task.

### REGIONS RELEVANT TO SOMATOSENSORY PROCESSING

This study demonstrated the utility of DTI for investigating neural connectivity. For the brain of the naked mole-rat, this strength can be utilized to examine the networks of the midbrain, especially the superior colliculus (SC), because the unique functions of the naked mole-rat SC have been well studied. We focused on the SC because it has an important role in processing visual information, especially eye and/or head and body movements. The superficial layers are responsible for receiving and processing visual inputs (May, 2006) and are severely atrophied in naked mole-rats (Crish et al., 2006b) and blind mole rats (Cooper et al., 1993). Furthermore, the SC receives somatosensory information from tactile hairs and projects to the facial nucleus (Izraeli and Porter, 1995; Miyashita and Mori, 1995; Hattox et al., 2002). Processing of somatosensory, auditory, and inhibitory inputs in the intermediate and deeper layers is integrated with visual information to acquire spatial information and elicit a concerted action (Drager and Hubel, 1975). Given that the reduction of SC volume in naked mole-rats is considered to be the result of atrophy of the superficial layers (Crish et al., 2004), it seems that their SC is specialized for somatosensory oriented behaviors, in contrast to other sighted rodents in which the SC functions in visual processing (Crish et al., 2006b). This implies that the anatomical structure of the SC in naked mole-rats is unique.

Naked mole-rats have a highly developed somatosensory system to detect external stimuli. In mammals, a major source of somatosensory information comes from the facial vibrissae. Naked mole-rats also have these vibrissae on their face, but in addition they have approximately another 40 tactile hairs on each side of their body, which have similar functions to the facial vibrissae (Crish et al., 2003). Information from the tactile hairs can guide accurate and appropriate behaviors (Crish et al., 2003), and the SC is involved in vibrissae orienting behaviors (Crish et al., 2006b). Since there is a tight connectivity of neural circuits between the afferent nerve from body and the SC, the connectivity of the SC may show unique features. For example, it is of interest whether the connection between superficial layers and the optic nerve can be visualized. In future studies, it is worth probing the neural network around the midbrain in detail by applying advanced diffusion tractography such as diffusion spectrum imaging (Wedeen et al., 2012) combined with neural tracing.

As somatosensory occupation was suggested from observation of cortical thickness, the dominance of the somatosensory system has also been observed from previous studies (Crish et al., 2003; Park et al., 2003), with inputs derived not only from unique tactile hairs, but also from the teeth (Catania and Remple, 2002). In addition to general uses of the teeth such as in digging, the huge teeth of the naked mole-rat have another role in aggressive facial behaviors (Sherman et al., 1991), which may be associated with hierarchy formation (Brecht and Freiwald, 2012). Queens use aggressive facial behaviors to govern their society (Reeve, 1992; Clarke and Faulkes, 1997), and members of this society may in turn show morphological differences depending on their social status. Given that neural changes might occur during the shift from non-breeder to breeder (Holmes et al., 2007), it is important to examine morphological changes in structures throughout the brain during a promotion of social status (i.e., from non-breeder to breeder).

In human subjects, analyses of subtle differences in brain anatomy such as sex difference (Good et al., 2001) or life-span brain development (Giorgio et al., 2010a,b; Groeschel et al., 2010) have been examined using VBM. In small animals subjects, as was conducted with naked mole-rats in this study, the establishment of *in vivo* MRI combined with the atlas in stereotaxic coordinates will enable to apply VBM with a statistical atlas in future. Following development of VBM for naked mole-rats allows us to evaluate longitudinal changes by focusing the process of sexual maturation, which is a prominent characteristic of the exceptional eusocial mammal.

## CONCLUSION

Naked mole-rats have a number of as yet poorly characterized interesting features, and this atlas as well as the *in vivo* imaging techniques will play an important role in their analysis by using anatomical information from various different perspectives. We detected atypical development of the visual system via a DTI and differences in cortical distribution between the naked mole-rat and the mouse, which might result from altered development of the somatosensory system relative to the visual system. Further examination of the sensory system by MRI would be valuable as it could be used to non-invasively track visual system development as well as to precisely localize cortical distribution in specific subjects, and could be coupled with the construction of VBM based on the present atlas and *in vivo* imaging, for the analysis of morphological differences of social status in a hierarchical classification.

## Conflict of Interest Statement

The authors declare that the research was conducted in the absence of any commercial or financial relationships that could be construed as a potential conflict of interest.

## AUTHOR CONTRIBUTIONS

Fumiko Seki and Keigo Hikishima conducted research; Sanae Nambu, Kazuo Okanoya, Hirotaka J. Okano, Erika Sasaki, and Hideyuki Okano provided essential materials. Fumiko Seki, Keigo Hikishima, and Kyoko Miura wrote the paper; Kyoko Miura and Hideyuki Okano had primary responsibility for final content. All authors read and approved the final manuscript.

## Footnotes

1 http://www.trackvis.org

2 http://nifti.nimh.nih.gov/

3 http://131.113.177.229/mri/

## Abbreviations

3D: three-dimensional
cc: corpus callosum
CT: computed tomography
DEC: direction-encoded color
DTI: diffusion tensor imaging
LGN: lateral geniculate nucleus
MRI: magnetic resonance imaging
RARE: rapid acquisition with relaxation enhancement
SC: superior colliculus
T_2_WI: T_2_-weighted image
VBM: voxel based morphometry.
